# Management of acute metabolic decompensation in maple syrup urine disease: guidance based on international clinical practice

**DOI:** 10.1186/s13023-026-04418-y

**Published:** 2026-06-06

**Authors:** Aude Servais, Marie Therese Abi-Wardé, Jean-Baptiste Arnoux, Alberto Burlina, Francois Feillet, Vincenzo Giordano, Nicola Longo, Sufin Yap

**Affiliations:** 1 Department of Adult Nephrology and Transplantation, Necker- Enfants Malades University Hospital, APHP, Imagine Institute, 149 Rue de Sèvres, 75015 Paris, France; 2 Reference Center for Hereditary Metabolic Diseases, Paris, France; 3 Department of Paediatric Neurology and Metabolic Disorders, University Hospital of Strasbourg, Strasbourg, France; 4 Division of Inherited Metabolic Diseases, University Hospital of Padua, Padua, Italy; 5 Reference Centre for Hereditary Metabolic Diseases, Paediatric Unit, University Hospital of Nancy, Nancy, France; 6 Recordati Rare Diseases, Medical Affairs, Puteaux, France; 7 Division of Clinical Genetics, Department of Human Genetics, University of California Los Angeles, Los Angeles, CA USA; 8 Department of Inherited Metabolic Diseases, Sheffield Children’s Hospital, Sheffield, UK

**Keywords:** Acute metabolic decompensation, Branched-chain amino acids, Guidelines, Leucine, Maple syrup urine disease, Treatment

## Abstract

**Background:**

Maple syrup urine disease (MSUD) is an autosomal recessive inborn error of metabolism caused by a deficiency of branched-chain ketoacid dehydrogenase, the enzyme involved in the second step of branched-chain amino acid catabolism. Of the three branched-chain amino acids (leucine, valine, and isoleucine), accumulation of leucine is the predominant factor causing acute metabolic decompensation in patients with MSUD.

**Main body:**

In February 2025, eight expert physicians met to discuss the management of acute metabolic decompensation and propose recommendations after literature review (four guidelines and 20 other articles of interest). A practical clinical algorithm was established. Newborn screening was acknowledged to be a successful method of diagnosing MSUD at birth, facilitating early intervention to prospectively manage MSUD and reduce the frequency and severity of acute metabolic decompensation, although it was noted that infants with severe MSUD often present with acute metabolic decompensation before being diagnosed. The experts also identified several barriers to and gaps in the management of acute metabolic decompensation, and MSUD more generally, proposing potential actions to improve clinical outcomes. Acute metabolic decompensation requires prompt, effective treatment by a multidisciplinary team to ensure that circulating plasma leucine levels are rapidly reduced without causing complications (particularly cerebral edema). Where available, intravenous branched chain amino acid-free solutions (e.g., Maapliv, now approved in Europe) may represent an important treatment option. Adequate resources (treatments, laboratory services, dialysis units) are essential for effective management of acute metabolic decompensation. Liver transplantation is an accepted viable option for the long-term prevention of acute metabolic decompensation in eligible patients. Research is ongoing into new treatment options for MSUD, such as gene therapy.

**Conclusion:**

Optimal management of acute metabolic decompensation in patients with MSUD requires prompt, effective treatment to reduce leucine levels without causing complications. A ready-to-use branched chain amino acid-free intravenous solution has been recently approved in Europe and research into new treatment options is ongoing.

**Supplementary Information:**

The online version contains supplementary material available at 10.1186/s13023-026-04418-y.

## Background

Maple syrup urine disease (MSUD) is a rare, autosomal recessive, inherited disorder, caused by biallelic mutations in the *BCKDHA*, *BCKDHB*, *DBT*, or *PPM1K* genes [1]. The estimated incidence of MSUD is 1 per 185,000 live births worldwide [2], 2 per 201,000 in Italy [3], 1 per 200,000 in the United States (US) [4], 1 in 147,975 in Ireland [5], and 3 in 626,000 in France [6]. Similar to other metabolic conditions, MSUD is detected either through clinical presentation or newborn screening (NBS); NBS of MSUD relies on the detection of specific amino acids (AAs; leucine, isoleucine, or valine), which are known collectively as branched-chain AAs (BCAAs) [4].

In contrast to classic organic acidurias, such as propionic and methylmalonic acidemias, which are mainly caused by accumulation of organic acids and subsequent mitochondrial toxicity [7], MSUD is caused by a deficiency of branched-chain ketoacid dehydrogenase (BCKD), the enzyme necessary for the second step in the catabolism of the three branched-chain ketoacids derived from the BCAAs [2, 8]. BCKD deficiency leads to elevated circulating plasma levels of all three BCAAs, which have specific neurotoxicity. Leucine is present at the highest levels [9], with lesser, but still substantial, accumulation of valine and isoleucine [10]. Alloisoleucine is a sensitive, specific, and reliable diagnostic marker of all forms of MSUD; this derivative of isoleucine is almost undetectable under normal physiological conditions. Diagnostic confirmation of MSUD is achieved with testing of the *BCKDHA*,* BCKDHB*,* DBT* and *PPM1K* genes [9].

Acute metabolic decompensation (AMD) in MSUD is caused by the accumulation of BCAAs, especially leucine, usually triggered by excess protein intake or endogenous protein catabolism because of infection, surgery, injury, fasting, or a significant dietary change [2, 11]. Guidelines for management of AMD in patients with MSUD recommend monitoring of BCAA levels, cessation of protein intake, administration of carbohydrate- and fat-based calories, maintaining hydration, preventing dilutional electrolyte imbalance by avoiding hypo-osmolar solutions, up titration of BCAA-free feeds (referred to henceforth as BCAA-free solutions or mixtures) and, if necessary, dialysis [9, 12–14]. As therapy continues, isoleucine and valine levels decrease, leading to a deficiency of these BCAAs; therefore, patients require isoleucine and valine supplementation. Care should be taken to avoid excessive fluid administration, to minimize the risk of hyponatremia and subsequent brain edema.

Despite these recommendations, there remain several barriers to and gaps in the management of AMD in patients with MSUD. To address these issues, a meeting of experts in MSUD (the authors of this article) was conducted to examine the available literature and discuss clinical experiences. Based on these discussions, this article provides an overview of important themes in the management of MSUD, as well as detailed discussion and practical recommendations for how AMD should be treated in clinical practice, including a practical clinical algorithm. Finally, this expert opinion proposes strategies to optimize outcomes for patients with MSUD and their families/caregivers.

## Methodological approaches

### Literature search

A search of PubMed was conducted on 18 December 2024 for articles published since 2010 on MSUD using the terms ‘*maple syrup urine disease*’ OR ‘*MSUD*’. It was considered that this search methodology would capture pertinent and recent research in the area. The articles were filtered manually to obtain the most relevant information for discussion at the meeting and relevant articles were evaluated by the expert authors.

Of the 645 results obtained from the literature search, 24 key publications that focused on the management of acute decompensation in MSUD were identified from the title and abstract of each article. Guidelines, reviews, clinical interventional and non-interventional studies were considered, but case reports were excluded. The identified publications comprised 17 clinical studies (including one cross-sectional study; summarized in the table in Additional file 1) [5, 15–30], as well as four published guidelines for the management of MSUD from the USA [12], Saudi Arabia [13], France [9], and Iran [14] (Table 1) and three review articles [11, 31, 32]. These publications were used to inform discussions by the experts at the meeting.

**Table 1:** Summary of guidelines for the management and prevention of AMD in patients with MSUD

Guideline, country (year)	Laboratory testing	Treatment	Other considerations
Diagnosis and management guidelines, Iran (2025) [14]	*For diagnosis*: • Urine ketones • Blood chemistry • Blood gas *Monitoring should include*: • Blood glucose every 4–6 h with a glucometer • Blood AAs, serum phosphate, and magnesium every 12–24 h • Lipase, amylase and transaminases every 24–48 h • Blood sodium every 12–24 h • Urine ketones with each urination	• Stop natural protein intake for 24–48 h (may be increased to 72 h) • Hydration and calorie supply o 10–12.5% dextrose in normal saline o IV BCAA-free solution 2–3.5 g/kg/d, if available o Isoleucine and valine supplements o NGT feeding if patient can tolerate oral administration • Correct metabolic abnormalities o Blood glucose levels should be 100–160 mg/dL o If hyperglycemia occurs, start insulin infusion at the rate of 0.02–0.15 IU/kg/h, which also helps to improve anabolism • Toxin removal o Consider HD or PD if leucine > 1100 µmol/L and/or there is rapid onset of neurological symptoms • Finding the underlying cause of the metabolic crisis o Nutritional support is ineffective if the underlying cause is not addressed • Cofactor treatment o Thiamine 50–200 mg/d • Minimize complications o Manage infection (candida), trauma, dehydration, fever, nausea, vomiting • Avoid ketorolac, glucocorticoids, vasoactive catecholaminergic agents, and drugs that affect renal perfusion. • Intermediate MSUD can be treated with phenylbutyrate	• Reintroduction of intact protein (or complex AA mixtures) • Identify underlying cause(s) of metabolic crisis, cofactor treatment, and minimize complications
Haute Autorité de Santé National Protocol, France (2021) [9]	• Leucine level	*On diagnosis of AMD*: • Immediately initiate treatment with a BCAA-free solution via oral/NGT feeding; an IV formulation should be used when the nasogastric route is not tolerated • Leucine level < 400 µmol/L (+ risk of AMD) or 400–750 µmol/L o Initiate ‘emergency diet’ o Maintain/increase BCAA-free feeds to ≥ 2 g/kg/d for children and 1–1.5 g/kg/d for adults o Supplement valine and isoleucine for 24–48 h, if available • Leucine level > 750 µmol/L o Hospitalize patient o Carbohydrates and lipids via IV or NGT administration (depending on clinical severity and digestive signs e.g., vomiting) o Maintain BCAA-free intake PO or via NGT o Supplement valine and isoleucine for 24–48 h, if available • Leucine level > 1100 µmol/L (severe AMD) o Initiate close clinical monitoring o Measure leucine levels daily o Carbohydrates and lipids via IV or NGT route (depending on clinical severity and digestive signs e.g., vomiting) o Maintain BCAA-free intake PO or via NGT o Dialysis or hemofiltration depending on neurologic signs, clinical situation (age, aggravating factors, kinetics of leucine levels) • Leucine level > 1500 µmol/L or clinical signs indicating vital risk o Admit to ICU o Measure leucine levels daily o Carbohydrates and lipids given IV or via NGT (depending on clinical severity and digestive signs e.g. vomiting) o Maintain BCAA-free intake PO or via NGT o Dialysis or hemofiltration depending on neurologic signs, clinical situation (age, aggravating factors, kinetics of leucine levels) *Target leucine level and discharge criteria*: • Continue treatment until leucine levels are < 150 µmol/L; if the level of < 150 µmol/L remains after 24 h, continue diet at 100% leucine tolerance; check the patient’s appetite and caloric intake before discharge	• Reintroduction of intact protein (or complex AA mixtures)
Acute Illness Protocol, Saudi Arabia (2018) [13]	• Chemistry panel for sodium, bicarbonate levels, and anion gap (to evaluate for increased anion gap metabolic acidosis and hyponatremia) • Venous blood gas • Plasma AA profile • Urinary ketones • Workup for infections (CBC, cultures, chest X-ray, urinalysis)	• Withhold intact protein intake if AMD risk is based on clinical status, ketosis, or plasma AA levels • BCAA-free solution 2–3.5 g/kg/d (with calculated amounts of valine and isoleucine) via oral/NGT feeding in the first instance, with IV formulations used when NGT feeding is not possible (e.g., aspiration or feeding intolerance) • Increase caloric intake by 25–50% above recommended dietary allowance; starting dose of lipids is 1–2 g/kg/d • Supplement with thiamine 50–200 mg/d • Use IV fluids cautiously in non-comatose patients to prevent cerebral edema; if cerebral edema does occur (monitor by CT and/or MRI), manage by elevating the head of the bed, hyperventilation, hypertonic saline and mannitol • Insulin may be required to maintain normoglycemia • HD to be considered in conjunction with a nephrologist	–
Nutrition Management Guidelines, US (2014) [12]	• Urine ketones • Blood gas	• Acute dietary treatment when at risk of AMD due to trauma, surgery, illness, or inappropriate dietary intake • Monitor biochemical and clinical status closely • Prevent catabolism and accumulation of endogenous BCAAs and BCKAs • Provide adequate BCAA-free exogenous protein, energy, fluid, and valine and isoleucine to promote anabolism • Achieve the recommended BCAA levels by administering BCAA-free solution via oral/NGT feeding in the first instance, with IV formulations used when NGT feeding is not possible • Thiamine supplementation • Pregnancy and the postnatal period; increased energy needs should be supported during pregnancy; monitor carnitine levels and supplement, if required; nutritional counselling if women experience nausea and vomiting during pregnancy; monitor breastfeeding women closely • LT: evaluate eligibility on a case-by-case basis; candidates should have good dietary management of BCAAs; prevent AMD post-surgery	• Reintroduction of intact protein (or complex AA mixtures)

AA, amino acid; AMD, acute metabolic decompensation; BCAA, branched-chain amino acid; BCKA, branched-chain keto acid; CBC, complete blood count; CT, computed tomography; d, day; h, hour; HD, hemodialysis; IV, intravenous; ICU, intensive care unit; LT, liver transplantation; MRI magnetic resonance imaging; MSUD, maple syrup urine disease; NGT, nasogastric tube; PD, peritoneal dialysis; PO, orally; US, United States

The abovementioned guidelines covered most areas of MSUD diagnosis and management [9, 12–14], but additional studies were identified that addressed the following specific topics: diagnostic testing for MSUD [13, 31]; the management of AMD [11, 15, 16, 19, 25, 29]; comparing the suitability of oral, nasogastric, and parenteral BCAA-free treatments [16, 17, 19, 25–27]; valine and isoleucine supplementation [16, 21, 27, 30]; plasma leucine levels and the rate at which they decrease [5, 15, 16, 20, 21, 23–25, 28–30]; hemodialysis (HD) and peritoneal dialysis (PD) [5, 18, 20, 24]; BCAA-free solutions as surgical prophylaxis [19, 25]; liver transplantation (LT) to treat MSUD [11, 23, 28]; disease and treatment complications [11, 17, 26–28, 32]; long-term dietary management of MSUD [5, 11, 26]; neurologic, psychiatric, and psychologic effects of living with MSUD [15, 22, 23, 28, 31, 32]; quality of life (QoL) of patients and their families/caregivers [21, 22]; and the need for new interventions to improve MSUD management [5, 18, 22, 23, 26, 28, 29, 31].

In addition to the publications identified by the search, the authors’ knowledge of MSUD was used to identify other relevant articles.

### Expert meeting

A virtual meeting of experts in pediatric and adult metabolic diseases from across Europe (United Kingdom [UK], France, and Italy) and the USA who routinely manage patients with MSUD was organized in February 2025. At the meeting, the results of the literature search on the management of AMD in patients with MSUD were evaluated and discussed. The aims of the meeting were to review the current treatment options for AMD, and to identify: (1) any gaps in the recommendations that need to be addressed; (2) any aspects of healthcare systems that may represent a barrier to optimal treatment; and (3) how clinical practice could be improved to ensure patients with MSUD receive adequate care to optimize clinical outcomes.

Clinical practice recommendations were developed in April 2026 according to the European Reference Networks guidelines [33] and discussed by the experts according to their level of agreement after literature review. Evidence was graded according to the American Academy of Pediatrics guidelines as follows: Level A = well-designed, randomized controlled trials (RCTs); Level B = RCTs with some limitations and substantial consistent evidence from observational studies; Level C = few observational studies; Level D = expert opinion, case reports, or first-principle–based reasoning; and Level X = exceptional situations in which validating studies cannot be performed but there is a clear preponderance of benefit or harm [34]. After discussion, the experts voted on their level of agreement for all grading and strength of recommendation using a 5-point scale (1 = strongly disagree, 2 = disagree, 3 = neither agree nor disagree, 4 = agree, 5 = strongly agree), according to the Delphi method [35]. It was decided a priori that ≥ 70% agreement for each statement was required; statements with < 70% agreement would be re-graded after discussion among the expert authors, and voting would be repeated. A summary of the expert authors’ recommendations, along with the grade and strength for each statement, is provided in Table 2.

**Table 2:** Summary of expert authors’ recommendations for the management of AMD in patients with MSUD

Recommendation	Grade	Strength
1. Plasma testing for AAs is the most reliable diagnostic test for MSUD, accurately quantifying levels of alloisoleucine, a pathognomonic marker of all forms of MSUD.	Level B	Strong
2. The general principles of AMD management are: (1) Testing of plasma BCAAs; (2) Discontinuation of protein intake from food sources; (3) Administration of oral, nasogastric, or parenteral BCAA-free solutions; (4) Isoleucine and/or valine supplementation; (5) Increasing caloric intake (IV, oral and/or enteral route); (6) Correction of underlying metabolic abnormalities.	Level B	Strong
3. Initially treat patients with BCAA-free solutions given orally or via NGT, with the latter method preferred in patients with anorexia without concurrent diarrhea or vomiting. If NGT feeding is not tolerated because of vomiting, the IV route should be used.	Level C	Moderate
4. Administration of IV BCAA-free solutions is recommended in emergencies, where plasma leucine levels are particularly high and/or when signs of metabolic decompensation are present.	Level C	Moderate
5. During severe AMD (plasma leucine > 1500 µmol/L in adults or > 1100 µmol/L in children, and/or rapid onset of neurologic symptoms), dialysis should be considered to rapidly and effectively remove leucine and toxins from the blood, even before the availability of plasma leucine levels.	Level X	Strong
6. High plasma leucine levels are a major risk factor for cerebral edema. Ensuring that fluids are restricted and that serum sodium is in the high-normal range will reduce the risk of developing acute cerebral edema.	Level X	Moderate
7. Long-term management of chronic MSUD requires a low-protein diet, supplemented with a BCAA-free solution.	Level B	Strong
8. Clinicians should consider offering LT as a viable treatment option in patients with severe MSUD to improve long-term metabolic control and to prevent the emergence of AMD.	Level B	Moderate

AA, amino acids; AMD, acute metabolic decompensation; BCAA, branched-chain amino acid; HD, hemodialysis; IV, intravenous; LT, liver transplantation; MSUD, maple syrup urine disease; NBS, newborn screening; NGT, nasogastric tube; PD, peritoneal dialysis

## Diagnosis of MSUD

### Forms of MSUD

MSUD is categorized into two main forms: classic and variant [14]. The classic form presents with acute neurologic signs in the newborn (disturbed consciousness, slow movements characteristic of pedaling and boxing, axial hypotonia, peripheral hypertonia, and characteristic maple syrup odor in the cerumen and urine) and is life-threatening if left untreated [2, 11]. The variant forms of MSUD (in increasing rarity) are intermittent, intermediate, and thiamine-responsive, and are categorized based on the severity of the enzyme deficiency and clinical symptoms [2, 14]. Typically, symptoms of the intermittent and intermediate forms manifest before the age of 7 years and continue to occur thereafter [2, 14]. Ideally, the MSUD assessment should be made at a specialist center for inherited metabolic diseases [9].

### Newborn testing

The impact of early diagnosis by NBS on mortality and neurocognitive outcomes cannot be underestimated, although infants with severe MSUD often present with AMD before they are diagnosed by NBS. Most NBS programs test for the biomarker XLE, an aggregate of leucine, isoleucine, alloisoleucine, and hydroxyproline [11], that cannot be individually quantified with standard NBS. However, NBS may sometimes miss intermediate forms of MSUD because XLE levels may be only moderately elevated or even normal [36]. A second-tier test may be conducted following an abnormal XLE result with chromatographic separation of leucine, isoleucine, alloisoleucine, and hydroxyproline from the same blood spot [2]. Second-tier testing can reliably distinguish clinically significant MSUD from hydroxyprolinemia (which is benign and does not require intervention) [2]. Plasma testing for AAs is recommended as the most reliable diagnostic test for MSUD for accurately quantifying levels of alloisoleucine, a pathognomonic marker of all forms of MSUD.

Diagnosis of the intermediate form of MSUD with NBS remains problematic because leucine levels are not always elevated during the newborn period. It depends on the timing of specimen collection, the specific laboratory threshold, and the availability of second-tier testing. In France, for example, if XLE levels are > 500 µmol/L during NBS, the neonate is immediately referred to a specialist metabolic center because of the high risk of coma in cases of classic MSUD. If XLE levels are 300–500 µmol/L, the risk of neonatal coma is considered low; therefore, the neonate will undergo a confirmatory test on a separate sample, a second-tier test with liquid chromatography (as described above), or measurement of plasma AA levels within 48 h after the first NBS to determine whether the intermediate form of MSUD is present [8, 37].

**Table:** 

**Clinical Practice Point 1:**
Plasma testing for AAs is the most reliable diagnostic test for MSUD for accurately quantifying levels of alloisoleucine, a pathognomonic marker of all forms of MSUD.
**Level B; Strong recommendation**

## Treatment of AMD in MSUD

**Fig. 1:**
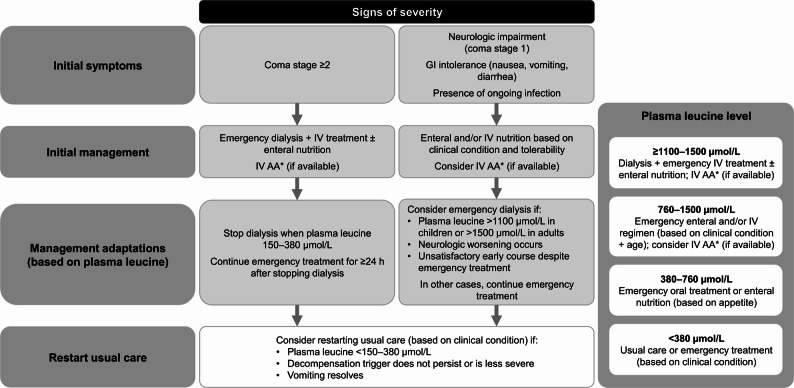
Treatment algorithm for the management of AMD in patients with MSUD. *Valine + isoleucine supplementation, initiated ≤ 48 h after starting emergency treatment. AA, amino acid; AMD, acute metabolic decompensation; GI, gastrointestinal; IV, intravenous; MSUD, maple syrup urine disease

Treatment options for AMD depend on clinical factors (age, plasma leucine level, neurologic status, gastrointestinal intolerance, and the presence of on-going infection) and available healthcare resources, specifically the availability of intravenous (IV) BCAA-free solutions. Figure 1 provides a practical clinical algorithm for the management of AMD based on plasma leucine levels and signs of severity.

Based on current guidelines (Table 1), the following general principles are recommended for AMD management: (1) testing of plasma BCAAs; (2) discontinuation of protein intake from food sources; (3) administration of oral, nasogastric, or parenteral BCAA-free solutions at 1.0–3.5 g/kg/day in children (according to the authors’ experience: 2.0–3.5 g/kg/day by nasogastric tube [NGT] or 1.0–2.0 g/kg/day by parenteral route) or 1.5–2.0 g/kg/day in adults; (4) isoleucine and/or valine supplementation; (5) increasing caloric intake by 25–50% above the European Food Safety Authority recommended dietary allowance [38] by giving emergency infusions of lipids (starting dose of 1–2 g/kg/day or 2–3 g/kg/day in neonates according to authors’ experience) and dextrose (20% with electrolytes); (6) correction of underlying metabolic abnormalities (insulin may be required to manage hyperglycemia; maintain normal-high sodium intake and potassium balance with supplements); (7) HD or PD when plasma leucine levels are > 1100 µmol/L in children or > 1500 µmol/L in adults or if neurologic symptoms are present [9, 12–14]. In addition, cofactor treatment with thiamine 50–200 mg/day should be initiated in thiamine-responsive patients, and precipitating factors, such as infection, trauma, dehydration, fever, nausea and/or vomiting, and fasting, should be managed [9, 12–14]. The current guidelines also outline the laboratory tests to be performed and how to manage complications, which may be adapted according to the local availability of drugs and medical foods.

**Table:** 

**Clinical practice point 2**
The general principles of AMD management are:
(1) Testing of plasma BCAAs;
(2) Discontinuation of protein intake from food sources;
(3) Administration of oral, nasogastric, or parenteral BCAA-free solutions;
(4) Isoleucine and/or valine supplementation;
(5) Increasing caloric intake (IV, oral and/or enteral route);
(6) Correction of underlying metabolic abnormalities.
**Level B; Strong recommendation**

### BCAA-free solutions

Since leucine is the most abundant BCAA in natural protein, it accumulates during AMD in patients with MSUD. Therefore, natural protein intake should be restricted [2, 11, 19, 28] and increased doses of leucine-free solutions must be administered during AMD. At present, oral BCAA-free solutions are commercially available (which can also be administered via NGT) and, in some countries (e.g., France and Italy), IV BCAA-free solutions are also available. However, the authors note that IV BCAA-free solutions are not readily available in all countries/locations, and given the rarity of MSUD, these solutions may expire before they are used.

The expert authors agreed that oral or NGT feeding should deliver calories and BCAA-free solutions. However, clinicians cannot be sure of the exact quantity of AA intake or absorption with oral administration, especially in patients with gastrointestinal symptoms [2]. Although less effective in reducing plasma leucine levels than IV formulations, oral BCAA-free solutions are preferred in patients with mild metabolic decompensation or in those recovering after severe AMD.

Initial treatment with BCAA-free solutions given orally or via NGT is recommended, with the latter method preferred in patients with anorexia without concurrent diarrhea or vomiting; if NGT feeding is not tolerated because of vomiting, the IV route should be used. IV administration is also indicated when NGT feeding cannot be restored in patients who have been vomiting for ≥ 24 h. Another consideration in selecting the administration route is patient preference. For example, in clinical practice, adults often prefer not undergoing NGT insertion, in which case the IV route may be preferable. Administration of IV BCAA-free solutions is recommended in emergencies, where plasma leucine levels are particularly high and/or when signs of metabolic decompensation are present [16, 19, 25]; these IV solutions are administered continuously over several days until plasma leucine levels normalize [16, 25]. Scott and colleagues have suggested that a factor of 0.5 (i.e., plasma leucine levels decreasing by approximately 50% over each 24-h period) may be considered a good estimate of the decline in plasma leucine levels when using nutritional support and a BCAA-free feed during clinical management of MSUD [26].

**Table:** 

**Clinical practice point 3**
Initially treat patients with BCAA-free solutions given orally or via NGT, with the latter method preferred in patients with anorexia without concurrent diarrhea or vomiting; if NGT feeding is not tolerated because of vomiting, the IV route should be used.
**Level C; Moderate recommendation**
**Clinical practice point 4**
Administration of IV BCAA-free solutions is recommended in emergencies, where plasma leucine levels are particularly high and/or when signs of metabolic decompensation are present.
**Level C; Moderate recommendation**

#### Route of administration

Several comparative observational studies have evaluated the effectiveness of BCAA-free solutions administered via the oral or NGT versus IV route. A retrospective, single-center French study by Servais and colleagues assessed AMD in four adults, where 17 episodes treated with IV BCAA-free solution were compared with 18 previous episodes treated with BCAA-free solution administered via NGT [27]. This study reported a significantly greater overall decrease in plasma leucine levels with IV versus NGT administered BCAA-free solution (*p* = 0.0026). During the first 3 days of hospitalization, the decrease in plasma leucine levels was faster with IV-treated episodes than NGT-treated episodes; plasma leucine levels normalized (< 381 µmol/L) within 3.5 days in most IV-treated episodes, whereas plasma leucine levels decreased but did not normalize before hospital discharge with NGT-treated episodes [27]. This lack of normalization with NGT-treated episodes was caused by poor dietary compliance, with discharge before leucine normalization being more commonly observed with NGT- versus IV-treated episodes (seven vs. four cases). Notably, in four NGT-treated episodes, the patients refused enteral feeding by NGT and were instead treated with oral BCAA-free solutions, which led to the need for dialysis in one case due to neurologic symptoms. In contrast, no deterioration or adverse effects occurred with the IV-treated episodes [27].

A prospective, multicenter French study evaluated the effectiveness and safety of IV BCAA-free solutions for managing AMD (defined in this study as a plasma leucine levels of > 381 µmol/L) in 24 patients with MSUD (13 children and 11 adults) who experienced 102 efficacy-evaluable AMD episodes (22 in children and 80 in adults) [16]. The mean duration of continuous IV BCAA-free solution administration was 4.8 days in children (0.8–2.0 g/kg/day) and 3.8 days in adults (0.5–2.6 g/kg/day). Normalized plasma leucine levels (< 381 µmol/L) were reached in 82% of episodes in children and 84% of episodes in adults. The mean (standard deviation) time to plasma leucine level normalization was 3.0 (1.2) days in the overall population (2.7 [1.1] days in children; 3.0 [1.3] days in adults), which was significantly shorter than the predicted time to leucine normalization based on average leucine tolerance (5.1 days; *p* < 0.001). No treatment-related adverse events were reported [16].

Another retrospective study compared oral/enteral versus IV BCAA-free solutions in 54 patients with MSUD who experienced 126 AMD episodes in France and Germany (including eight episodes of treatment for surgical prophylaxis [6.3%]) [19]. Oral/enteral BCAA-free solutions were used in 94% of children and 44% of adults, while IV solutions were used in 85% of adults and 22% of children. Severe or critically elevated plasma leucine levels were more commonly seen among patients receiving IV versus oral/enteral solutions (25% vs. 7.2%). The mean (range) time to first leucine normalization (< 381 µmol/L) was 68.4 (6.1–334.3) h in the oral/enteral group and 95.6 (23.9–428.8) h in the IV group. The mean (range) time to episode resolution was 9.2 (2.0–35.0) and 7.4 (2.0–19.0) days in the respective groups, and the mean (range) duration of hospitalization was 6.6 (2.0–23.0) and 5.4 (2.0–14.0) days, respectively. Serious treatment-related nausea and vomiting occurred in the oral/enteral group, while no serious adverse events occurred in the IV group [19].

A retrospective, multicenter Spanish study investigated the effectiveness of IV BCAA-free solution as an emergency treatment for AMD (*n* = 5) or as surgical prophylaxis (*n* = 1; to prevent possible AMD due to fasting pre-surgery) in five pediatric patients with MSUD [25]. BCAA-free solution dosages were determined by age, with neonates and infants aged < 2 years receiving 2–3 g/kg/day and children aged > 2 years and adolescents receiving 1–2 g/kg/day. Two patients received a lower dose (≤ 1 g/kg/day) due to rapid improvement and resolution of vomiting that allowed administration of both oral and IV BCAA-free solutions. Treatment duration ranged from 3 to 8 days, with the exception of one patient, for whom IV solution was not available at admission, but after developing coma with cerebral edema at 12 h post-admission, IV BCAA-free solution treatment was immediately initiated and continued for 20 days thereafter. The duration of hospitalization ranged from 11 to 65 days. In all cases, plasma leucine levels normalized and symptoms had improved or resolved at hospital discharge. No adverse events were reported.

### Valine and isoleucine supplementation

During AMD, plasma BCAA levels rise, particularly leucine. With administration of BCAA-free solutions, plasma valine and isoleucine levels might decrease to below normal levels [30]. Valine and isoleucine promote protein anabolism and accelerate reduction of plasma leucine levels; therefore, most patients with AMD will require supplementation with limited amounts of valine and isoleucine after starting treatment with a BCAA-free solution. If valine or isoleucine supplements are not available, alternative forms can be successfully used [30].

When comparing NGT with IV administration of BCAA-free solutions, the expert authors noted that doses of valine or isoleucine supplementation are usually adjusted during daily clinical workup. They emphasized the importance of documenting levels of all BCAAs (including valine and isoleucine), to ensure that they are within the therapeutic range because a valine and/or isoleucine deficiency may limit anabolism and AMD can recur.

### Other pharmaceutic treatments

Since AMD may be associated with dehydration, it is vital to optimize hydration status with fluid replacements [29, 39].

Dextrose with appropriate sodium and potassium supplementation and lipids are administered to sustain caloric supply for 24 h after an AMD episode and insulin may be required to manage hyperglycemia [9, 14]. Thiamine 50–200 mg/day may also be used with restricted amounts of AAs because thiamine can increase the residual activity of BCKD [40].

In patients with mild forms of MSUD, phenylbutyrate may effectively decrease circulating BCAA levels by reducing phosphorylation/inactivation of the BCKD enzyme and enhancing the oxidation of BCAA [41–43]. Phenylbutyrate increases enzyme activity of branched-chain alpha-ketoacid dehydrogenase, which effectively decreases circulating BCAA levels despite adequate dietary protein intake in healthy individuals and in patients with MSUD [41]. In one study, oral phenylbutyrate 250 mg/kg/day was used as part of an emergency protocol in 10 patients receiving hypercaloric IV nutrition, continuous IV insulin, and an enteral BCAA-free solution, with valine and isoleucine supplementation initiated after 24 h of therapy [43]. In a case report of an infant with AMD, for whom HD was not available, phenylbutyrate 500 mg/kg/day was used as enteral therapy following a 24-h sodium phenylacetate/sodium benzoate infusion to continue normalization of plasma leucine levels [42]. The expert authors advised that response to phenylbutyrate treatment should be first tested in the absence of AMD before it can be considered for emergency use during AMD, and in patients with the mild form of MSUD and/or those who have missense variants in the gene responsible for MSUD. Phenylbutyrate can also be considered for the chronic treatment of MSUD to prevent or delay AMD.

AMD that is caused by infection or significant dietary changes should be treated appropriately in line with its cause: antibiotics should be administered to treat infection, and any dietary changes should be addressed promptly by the metabolic team responsible for patient care [11].

### Hemodialysis and peritoneal dialysis

During severe AMD (plasma leucine > 1500 µmol/L in adults or > 1100 µmol/L in children, and/or rapid onset of neurologic symptoms), dialysis should be considered to rapidly and effectively remove leucine and toxins from the blood [14], even before the availability of plasma leucine levels. The expert authors advised that, in their experience, HD is preferred over PD, irrespective of age. When performed promptly by an experienced and multidisciplinary team, HD is safe, effective, and potentially life-saving in most neonates with severe BCAA intoxication [20]. HD offers a rapid solution for any life-threatening metabolic crises; if HD is not available, PD may be used but clinicians should be aware that this is less effective than HD [24]. When the patient discontinues dialysis, it is important to maintain a high caloric intake to prevent a leucine rebound effect.

It is worth emphasizing that dialysis is an effective method for lowering circulating leucine levels, thereby promoting the clearance of leucine from the brain and preventing cerebral edema [24].

**Table:** 

**Clinical practice point 5**
During severe AMD (plasma leucine > 1500 µmol/L in adults or > 1100 µmol/L in children, and/or rapid onset of neurologic symptoms), dialysis should be considered to rapidly and effectively remove leucine and toxins from the blood, even before the availability of plasma leucine levels.
**Level X; Strong recommendation**

## Complications of treatment for AMD

During AMD, accumulation of leucine and other BCAAs in the brain can often lead to cerebral edema, which is further complicated by additional fluids received during treatment from BCAA-free solutions and glucose and lipid infusions [9]. The expert authors point out that high plasma leucine levels are a major risk factor for cerebral edema; ensuring that fluids are restricted and that serum sodium is in the high-normal range will reduce the risk of developing acute cerebral edema [44]. This is particularly important during recovery from AMD, when plasma leucine levels are being reduced. Diffusion of fluid into brain cells may ultimately lead to increased intracranial pressure and death in a patient who is recovering from high leucine levels [45].

In patients with severe cerebral edema, besides emergency treatment of AMD and/or dialysis, clinicians should ensure non-specific care of cerebral edema. This may include hypertonic (3%) sodium chloride 2–3 mEq/kg/dose, given together with AA supplementation [14]. In addition, mannitol 0.5–1 g/kg/dose and furosemide 0.5–1 mg/kg/dose should be administered [14]. Since valine and isoleucine boost anabolism, supplementation with these BCAAs may reduce the risk of cerebral damage, and should be continued as part of the emergency treatment of AMD [30].

Acute pancreatitis is another potential treatment complication, and should be managed according to the applicable guidelines [14]. Abi-Wardé and colleagues suggested that long-term leucine toxicity correlated with an increased AMD frequency and may lead to psychologic problems [15].

**Table:** 

**Clinical practice point 6**
High plasma leucine levels are a major risk factor for cerebral edema; ensuring that fluids are restricted and that serum sodium is in the high-normal range will reduce the risk of developing acute cerebral edema.
**Level X; Moderate recommendation**

## Long-term MSUD management: AMD prevention

The expert authors discussed two approaches to the long-term management of MSUD: (1) dietary management in patients with mild MSUD who are not at a high risk of AMD; and (2) LT in patients with severe MSUD.

### Dietary management

Long-term management of chronic MSUD requires a low-protein diet, supplemented with a BCAA-free solution. When treating adults, the main barrier to effective MSUD management is long-term patient adherence to this treatment. Most AMD can be prevented if patients adhere to their dietary management and are well-informed about implementing an emergency regimen to prevent catabolism.

Each patient has two or three types of diets: a well-day diet, which is the patient’s usual diet or 100% of their personal leucine tolerance; a sick-day diet, used in acute situations, where leucine intake is stopped for 24–48 h; and a semi-emergency diet or 50% of leucine tolerance [9]. Leucine tolerance is defined as the amount of leucine required for an individual to maintain plasma leucine levels within the target range, and to ensure optimal weight-bearing growth. While excessive leucine intake leads to intoxication, insufficient intake of BCAA-free solution prevents normal growth [9].

The sick-day protocol should be implemented during fever, surgery, any infectious disease, after excessive fasting time for any reason, or because of the possibility of a sudden increase in plasma leucine levels, acidosis, hypoglycemia, and/or decreased consciousness. This protocol usually entails increasing BCAA-free formula intake by 20%, decreasing leucine intake by 50–100%, increasing caloric intake through small but frequent feeds throughout a 24-h period, and reducing the duration of overnight fasting [11].

**Table:** 

**Clinical practice point 7**
Long-term management of chronic MSUD requires a low-protein diet, supplemented with a BCAA-free solution.
**Level B; Strong recommendation**

### Liver transplantation

Clinicians should consider offering LT as a viable treatment option in patients with severe MSUD to improve long-term metabolic control and to prevent the emergence of AMD [20, 46, 47]. A US study of 37 patients with classic MSUD reported 100% survival after LT, with stable BCAA metabolism after surgery and increased leucine tolerance (from 10 to 25 mg/kg/day to > 150 mg/kg/day) [46]. A single-center prospective Italian study also reported significant improvements in QoL following LT in 32 patients with intoxication-type inborn errors of metabolism, including six with severe MSUD [47]. However, patient willingness to undergo LT for MSUD seems to vary between countries.

Patients with MSUD who have good nutritional status are considered to be ideal candidates for LT (i.e., ideal body weight, good function of their other organs [lung, heart, bones]) compared with patients with other indications for LT, who may not tolerate surgery because of other comorbidities or risks (e.g., a 60-year-old smoker with liver cirrhosis and high alcohol consumption). Surgeons may perform a “domino” transplant, where the explanted liver from the patient with MSUD is used to save the life of another patient without MSUD. The expert authors emphasized that leucine, isoleucine, and valine blood levels usually stabilize within 6 h post-LT in patients with MSUD and remain as such with unrestricted protein intake [48]. Thereafter, these patients typically maintain a stable metabolic profile [49].

The expert authors highlight that there is a marked difference in clinical outcomes between patients who undergo LT versus dietary management. Patients with severe MSUD who underwent LT have been reported to have improved QoL and drastically reduced risk of subsequent AMD [20, 46]. Although long-term data are lacking, the experts note that LT reduces MSUD-related mortality; however, the limited number of donors remains a constraint when considering LT as a treatment option in patients with severe MSUD.

Although rare, it is possible that AMD may occur after LT [39, 50]. Therefore, patients should undergo lifelong metabolic follow-up post-LT, similar to patients with mild or intermittent MSUD. While the risk of post-LT complications is low, leucine levels may remain mildly elevated (< 2 x the upper limit of normal is acceptable). In the authors’ clinical experience, plasma leucine levels generally remained stable post-LT, even during times of illness, febrile episodes, or COVID infection.

In the experts’ opinion, the option of LT should be considered earlier in life among patients with severe MSUD, prior to reaching adulthood.

**Table:** 

**Clinical practice point 8**
Clinicians should consider offering LT as a viable treatment option in patients with severe MSUD to improve long-term metabolic control and to prevent the emergence of AMD.
**Level B; Moderate recommendation**

## Quality of life

The QoL in adolescents and adults with MSUD is impacted by social exclusion due to the restrictive diet that they must follow. The parents/caregivers of children with MSUD can also experience reduced QoL caused by the anxiety of an anticipated, potentially fatal AMD. Identifying factors that affect QoL will assist clinicians in planning effective social and psychologic interventions to improve QoL, and as such, enhance patient care and outcomes [22].

A study by Abi-Wardé and colleagues in 35 pediatric patients with MSUD (32 families) showed that 45% of families had difficulty coping with MSUD and that parental involvement was crucial in managing the disease [15]. Factors associated with poor QoL in parents were parent-related factors, such as being the father or the non-working parent, older age, having a greater anxiety level, and seeking greater social support rather than using positive thinking and problem-solving coping strategies [51]. Family- and disease-related factors also influenced parental QoL, including the presence of disease complications and a high number of hospital medical providers (as markers of disease severity), younger age of the patient, single-parent family, and lower family material wealth [51]. In children with inborn errors of metabolism, poorer QoL was associated with anxiety and behavioral problems, as well as parental anxiety, younger age at diagnosis, and having a disease that required an emergency diet [52]. Importantly, evidence suggests QoL improves drastically post-LT, because patients can reintegrate fully into society, and patients and their families are relieved of the psychologic burden that typically accompanies MSUD [46].

## Addressing the barriers and gaps in the management of MSUD

**Fig. 2:**
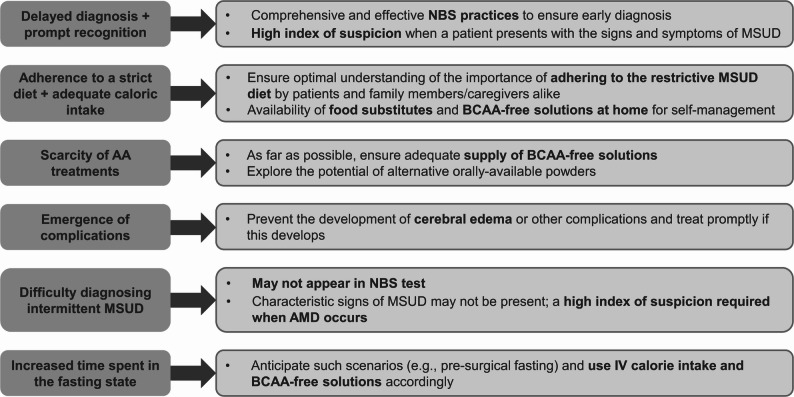
Issues associated with the management of AMD in patients with MSUD and potential actionable solutions. AA, amino acid; AMD, acute metabolic decompensation; BCAA, branched chain amino acid; IV, intravenous; MSUD, maple syrup urine disease; NBS, newborn screening

The expert authors considered the seven key barriers associated with the management of MSUD (Fig. 2): (1) obtaining an early diagnosis of MSUD; (2) prompt recognition of AMD; (3) patient adherence to a strict diet and adequate caloric intake; (4) the scarcity of IV BCAA-free solutions; (5) emergence of complications; (6) difficulty diagnosing intermittent MSUD; (7) and increased time spent in the fasting state. The range of potential actions that can be undertaken by the relevant stakeholders to address these issues are shown in Fig. 2.

Several gaps or unmet needs in the management of MSUD were also identified, and the experts proposed possible actions to address these gaps (Table 3). These were: (1) the need for improved patient management to preventing any further neurologic damage; (2) the scarcity of IV BCAA-free formulations; (3) a lack of national NBS programs; (4) limited availability of dialysis facilities; (5) insufficient involvement of family members in patient care; (6) limited availability of a skilled multidisciplinary team to manage patients with MSUD; (7) national health care systems under-resourced for treating patients with MSUD; (8) a lack of a registry for metabolic diseases; and (9) the lack of new, safe, and effective disease-modifying therapies for MSUD. Of note, the European Medicines Agency (EMA) recently approved Maapliv, an IV BCAA-free formulation for the treatment of patients with MSUD [53].

**Table 3:** Gaps in the management of AMD in patients with MSUD and potential actionable solutions

Gap	Potential solutions
Preventing any/further neurologic damage	• The ‘sick day protocol’ should be implemented in case of fever, surgery, any infectious disease, stress, the possibility of a sudden increase in leucine levels, acidosis, hypoglycemia, and/or a decrease in consciousness [14]
Absence or lack of homogeneity of national NBS programs	• Emphasize areas needing attention globally to achieve homogeneity of NBS over different countries • Increased numbers of screening laboratories
Availability of dialysis facilities	• PD can be used in resource-constrained areas if HD is not available [18]
Inadequate involvement of family members [22]	• Understanding and following the restrictive diet, and later, encouraging their children to adhere to the diet • Identifying the factors that affect QoL would be beneficial for improving patient care • Increase involvement of family members when evaluating outcomes and treatments • Planning effective social and psychologic interventions to enhance QoL
Policy on setting up MDTs at hospitals	• A multidisciplinary approach is essential, including emergency room physicians, nephrologists, psychologists, radiologists, geneticists, specialist physicians, dieticians, and a dialysis team [5]
Quality of national healthcare systems	• Implement robust policies that benefit health [31] • Engage relevant stakeholders to consider implementing policy changes, increasing the availability of treatments, and establishing NBS programs
Lack of a national registry for metabolic diseases	• More patient data that are available on a common registry would markedly benefit access and availability of MSUD data for clinical and epidemiologic research
New safe and effective disease-modifying therapies for MSUD are not yet available	• Greater research into the biochemistry and physiology of MSUD and its response to LT will afford key insights into the design of new therapies based on gene replacement or gene editing [28]

AMD, acute metabolic decompensation; BCAA, branched-chain amino acid; HD, hemodialysis; LT, liver transplantation; MDT, multidisciplinary team; MSUD, maple syrup urine disease; NBS, newborn screening; PD, peritoneal dialysis; QoL, quality of life

## Conclusions

The prompt and effective treatment of AMD in patients with MSUD is essential. Effective management of AMD should ensure that circulating plasma leucine levels are reduced as quickly as possible without causing complications, such as cerebral edema. Therefore, a skilled multidisciplinary team with specific resources (treatments, laboratory services, pharmacy services, and dialysis units) is required to achieve near normal BCAA levels. The expert authors agreed that the lack of readily available IV BCAA-free solutions for managing AMD was a primary barrier to improving outcomes in patients with MSUD. However, the recent EMA approval for the Maapliv IV BCAA-free formulation will overcome this barrier in patients with AMD. Encouraging suitable candidates to undertake LT is a viable option for the prevention of further AMD. Research is ongoing into new treatment options for MSUD, such as gene therapy.

## Supplementary Information

Below is the link to the electronic supplementary material.

Supplementary Material 1: Additional information from the 24 key publications on MSUD identified by the literature search can be found in the Word document named Additional file 1 [Clinical studies on the management of AMD and MSUD (in ascending order by year of publication)].

Supplementary Material 2

## Data Availability

Data sharing is not applicable to this article as no datasets were generated or analyzed during the review.

## Abbreviations

AA: Amino acid
AMD: Acute metabolic decompensation
BCAA: Branched-chain amino acid
BCKD: Branched-chain ketoacid dehydrogenase
EMA: European Medicines Agency
HD: Hemodialysis
IV: Intravenous
LT: Liver transplantation
MSUD: Maple syrup urine disease
NBS: Newborn screening
PD: Peritoneal dialysis
QoL: Quality of life
UK: United Kingdom
US: United States
