# Supplementary material for: Management of acute metabolic decompensation in maple syrup urine disease: guidance based on international clinical practice
Source: Orphanet J Rare Dis. 2026 Jun 6;21:279. doi: 10.1186/s13023-026-04418-y (PMC13471534; doi:10.1186/s13023-026-04418-y)
Supplement: Supplementary file 1 [file 13023_2026_4418_MOESM1_ESM.pdf]

Management of acute metabolic decompensation in maple syrup urine disease: Guidance based on international clinical practice

Authors: Aude Servais, Marie Therese Abi-Wardé, Jean-Baptiste Arnoux, Alberto Burlina, Francois Feillet, Vincenzo Giordano, Nicola Longo, Sufin Yap

Correspondence: Aude Servais (aude.servais@aphp.fr), Department of Adult Nephrology and Transplantation, Necker Hospital, Paris Descartes University, 149 Rue de Sèvres, 75015, Paris, France. Telephone: +33144495413. Fax: +33144495450.

Additional information

Clinical studies on the management of AMD and MSUD (in ascending order by year of publication)

Author, year of publication	Type of study (country), n	Key findings
**AMD episodes**		
Servais et al., 2013 [1]	Prospective (France), n=4	Enteral and IV BCAA-free solutions to treat AMD were compared. The decrease in mean leucine levels was higher during the 3 first days of IV vs NGT treatment. IV treatment was better tolerated and accepted than NGT insertion. High caloric diet and therapeutic levels of valine and isoleucine were maintained. IV administration of BCAA-free solution was a simpler, safer, and more efficacious option than NGT feeds, and should be available to all patients with AMD episodes and elevated leucine levels.
Bilgin et al., 2014 [2]	Retrospective, (Turkey), n=3	Leucine accumulation managed with PD and dietary and pharmacologic therapy. Two patients survived without neurologic impairment; the other had severe neurologic damage and died of sepsis at 9 months of age. Although extracorporeal dialysis should be the first dialysis treatment of choice in theory, PD was shown to prevent neurologic damage. If resources are limited, PD can be a life-saving procedure in some patients, if performed by skilled staff and under standard procedures.
Abi-Wardé et al., 2017 [3]	Retrospective (France), n=35	Data on academic achievement, psychologic care, family involvement, and biochemical parameters were evaluated. AMD (plasma leucine >380 μmol/L) was more frequent during the first year of life mainly due to infections and after 15 years, mainly due to infections and dietary noncompliance. Patients with more psychologic follow-up visits experienced more AMD episodes.
Scott et al., 2017 [4]	Retrospective (USA), n=15	Once dietary leucine was restricted, plasma leucine levels declined exponentially at a rate proportional to ~50% of the starting levels over each 24-hour period. Recovery of leucine levels was sensitive to clinical status and the event that triggered the leucine increase. Patients with an active URTI at the time of AMD recovered slowly. Patients with a history of dietary non-adherence also recovered slowly, though not as slowly as patients with an URTI. The authors suggest that targeting a drop of leucine levels by a factor of 0.5 per 24 hours can be a good estimate to reach treatment goals.
Strauss et al., 2020 [5]	Prospective (USA), n=176 [severe], n=6 [intermediate], n=2 [intermittent]	13,589 AA profiles were used to analyze leucine tolerance, AA homeostasis, estimated cerebral AA uptake, quantitative responses to anabolic therapy, and metabolic control after LT. Stringent dietary therapy is challenging to implement, fails to restore appropriate circulating AA concentrations, and does not fully prevent cognitive and psychiatric disabilities. LT eliminated the need for a restrictive diet and reduces the chance of life-threatening AMD. However, LT is associated with predictable morbidities and does not reverse pre-existing neurological damage.
Yildiz et al., 2020 [6]	Retrospective (Turkey), n=29	Clinicians should be aware of the risk factors for AMD and the signs and symptoms of a patient at risk of AMD. Variables independently associated with AMD were poor feeding, malaise, anion gap, and uric acid (which correlated with plasma leucine levels). Other indicators were vomiting, dehydration, neurologic signs, ketonuria, and ketoaciduria; plasma sodium, chloride, and glucose levels had little diagnostic value.
de Lonlay et al., 2021 [7]	Retrospective, observational (France, Germany),	126 AMD episodes were analyzed; IV BCAA-free solutions were used for gastric intolerance (33%), refusal of NGT feeding (31%), "emergency" (14%), or coma (8%), and as surgical prophylaxis (6%). Mean duration of hospitalization was 6.6 days with oral/NGT feeds and 5.4 days with IV feeds. Plasma leucine levels decreased by 69.3% and 71.3% in

Author, year of publication	Type of study (country), n	Key findings
	n=54	the oral/NGT- and IV-treated episodes, respectively. Mean time to resolution of AMD was 15.8 days with oral/NGT treatment and 7.7 days with IV treatment (P=0.008).
O' Reilly et al., 2021 [8]	Retrospective (Ireland), n=18	Plasma leucine outside the therapeutic range (≥400 µmol/L) was more frequently observed in infancy or during adolescence (due to infections, hormonal changes, noncompliance). Children require careful management during AMD in early childhood. The "sick day" regime (early implementation), strict diet, and dialysis as a rescue therapy can achieve a high level of metabolic control thus preventing AMD episodes. Two patients died in early childhood and one as an adult; 15/18 patients were alive and with essentially normal functioning (good clinical outcomes).
Ziadlou and MacDonald, 2022 [9]	Case series (Iran), n=5	Clinicians explored an alternative leucine-free powdered form of valine/isoleucine supplements. The higher the plasma leucine level, the more powder was administered per kg of body weight. Plasma leucine levels decreased within 3–4 days of starting dietary treatment. The rate of improvement in cognitive and motor development was faster than improvement in walking and speech development.
Alili et al., 2022 [10]	Prospective, observational (France), n=24	102 AMD episodes were analyzed; mean plasma leucine levels were ≥381 µmol/L at presentation. Doses of 0.5–2.6 g/kg/day of IV BCAA-free solution manufactured at the hospital itself were administered; mean time to normalization of plasma leucine was 2.7 days for children and 3.0 days for adults. The predicted mean time to normalization was 5.1 days. Thus, IV solutions may provide faster AMD resolution than oral/NGT administration. No AEs were reported.
Sanchez-Pintos et al., 2022 [11]	Retrospective, observational (Spain), n=6	5 AMD episodes were analyzed. One of the patients included in the study was at risk of AMD due to fasting pre-surgery. AMD treatment duration ranged from 3–8 days (with 1 patient treated for 20 days). Treatment with BCAA-free solutions resulted in decreased plasma leucine levels and resolution of symptoms in all cases; no AEs were reported. Readily-available IV BCAA-free preparations are recommended when NGT-feeding is not possible (coma, vomiting), especially in children.
Eisenstein et al., 2022 [12]	Retrospective (Israel), n=7	HD resulted in 87–97% reduction in plasma leucine levels; two consecutive HD sessions decreased mean ± SD plasma leucine levels from 2281 ± 631 to 179 ± 91 µmol/L (92.1% reduction).
Onal et al., 2024 [13]	Retrospective (Turkey), n=5	Evaluated the efficacy and outcomes of RRT in pediatric patients. HD showed significant reductions in leucine levels (median of 94.5%) and, therefore, plays a critical role in managing AMD.
**MSUD**		
Berat et al., 2021 [14]	Retrospective (France), n=32	Explored the use of feeding via a NGT during the first year of life in patients who also require dietary treatment. NGT-related complications were:<br>• Medical problems, such as vomiting (n=9), recurrent ear, nose and throat infections (n=5), cough (n=3), vasovagal syncope (n=1), and skin lesions related to NGT fixation (n=1)<br>• Difficulties inserting the NGT (n=28), frequent removal of the NGT (n=8), and rejection of NGT leading to an indication for gastrostomy (n=1)<br>• Complications related to pump or tubing failures (n=4, including 2 cases of severe hypoglycaemia with seizure and neurologic sequelae in 2 patients with glycogen storage diseases)

Author, year of publication	Type of study (country), n	Key findings
		Gastrostomy-related complications were:<br>• Worsening of feeding difficulties (according to the parents; n=7), skin lesions (n=2), local discomfort, aesthetic issues (n=2), leakage (n=1), vomiting (n=1), and frequent removal of the gastrostomy button (n=1)
Handoom et al., 2018 [15]	Non-randomized retrospective + prospective (Saudi Arabia), n=30	Significant decreases in plasma leucine and isoleucine levels (P=0.0008), but not in plasma valine levels (P=0.1148), were observed after patients were prescribed a diet that included low-protein medical foods; 36.7% of patients received valine supplements.
Mengler et al., 2024 [16]	Prospective, observational (Germany), n=33 [22 classic; 11 variant]	Initial peak plasma leucine levels increased linearly with age in patients with classic MSUD (median: 1712 mmol/L), but not in patients with variant MSUD. LT (median age 5.8 years) was not associated with better cognitive outcomes. Two patients were missed by NBS; thus, NBS may be highly sensitive for classic MSUD, but not the variant form.
Magdy et al., 2025 [17]	Cross-sectional (Egypt), n=36	QoL was evaluated in patients with MSUD and their families. The study provided a comprehensive account of their healthcare experiences and identified potential psychosocial issues. Group 1 (25% of patients) and Group 2 (75%) experienced good and poor QoL, respectively. Predictors of poor QoL were disturbed consciousness and seizures, while positive consanguinity, good dietary compliance, and good metabolic control were predictors of good QoL. It is important to address psychiatric issues with the families of patients with MSUD during follow-up care. Early diagnosis was also indicative of better QoL. Standardized MSUD-specific questionnaires are required.

AA, amino acid; AEs, adverse events; AMD, acute metabolic decompensation; BCAA, branched-chain amino acid; HD, hemodialysis; IV, intravenous; LT, liver transplantation; MSUD, maple syrup urine disease; NBS, newborn screening; NGT, nasogastric tube; PD, peritoneal dialysis; QoL, quality of life; RRT, renal replacement therapy; SD, standard deviation; URTI, upper respiratory infection; USA, United States of America.

References

1. Servais A, Arnoux JB, Lamy C, et al. Treatment of acute decompensation of maple syrup urine disease in adult patients with a new parenteral amino-acid mixture. J Inherit Metab Dis. 2013;36:939-44.

2. Bilgin L, Unal S, Gunduz M, Uncu N, Tiryaki T. Utility of peritoneal dialysis in neonates affected by inborn errors of metabolism. J Paediatr Child Health. 2014;50:531-5.

3. Abi-Wardé MT, Roda C, Arnoux JB, et al. Long-term metabolic follow-up and clinical outcome of 35 patients with maple syrup urine disease. J Inherit Metab Dis. 2017;40:783-92.

4. Scott AI, Cusmano-Ozog K, Enns GM, Cowan TM. Correction of hyperleucinemia in MSUD patients on leucine-free dietary therapy. Mol Genet Metab. 2017;122:156-9.

5. Strauss KA, Carson VJ, Soltys K, et al. Branched-chain α-ketoacid dehydrogenase deficiency (maple syrup urine disease): treatment, biomarkers, and outcomes. Mol Genet Metab. 2020;129:193-206.

6. Yıldız Y, Akcan Yıldız L, Dursun A, et al. Predictors of acute metabolic decompensation in children with maple syrup urine disease at the emergency department. Eur J Pediatr. 2020;179:1107-14.

7. de Lonlay P, Posset R, Mutze U, et al. Real-world management of maple syrup urine disease (MSUD) metabolic decompensations with branched chain amino acid-free formulas in France and Germany: a retrospective

observational study. JIMD Rep. 2021;59:110-9.

8. O'Reilly D, Crushell E, Hughes J, et al. Maple syrup urine disease: clinical outcomes, metabolic control, and genotypes in a screened population after four decades of newborn bloodspot screening in the Republic of Ireland. J Inherit Metab Dis. 2021;44:639-55.

9. Ziadlou M, MacDonald A. Alternative sources of valine and isoleucine for prompt reduction of plasma leucine in maple syrup urine disease patients: a case series. JIMD Rep. 2022;63:555-62.

10. Alili JM, Berleur MP, Husson MC, et al. Intravenous administration of a branched-chain amino-acid-free solution in children and adults with acute decompensation of maple syrup urine disease: a prospective multicentre observational study. Orphanet J Rare Dis. 2022;17:202.

11. Sánchez-Pintos P, Meavilla S, López-Ramos MG, García-Cazorla A, Couce ML. Intravenous branched-chain amino-acid-free solution for the treatment of metabolic decompensation episodes in Spanish pediatric patients with maple syrup urine disease. Front Pediatr. 2022;10:969741.

12. Eisenstein I, Pollack S, Hadash A, et al. Acute hemodialysis therapy in neonates with inborn errors of metabolism. Pediatr Nephrol. 2022;37:2725-32.

13. Önal HG, Nalçacıoğlu H, Özer I, Karalı DT. The efficacy and outcomes of renal replacement therapy in pediatric metabolic disorders. J Clin Med. 2024;13:6452.

14. Bérat CM, Roda C, Brassier A, et al. Enteral tube feeding in patients receiving dietary treatment for metabolic diseases: A retrospective analysis in a large French cohort. Mol Genet Metab Rep. 2021;26:100655.

15. Handoom B, Megdad E, Al-Qasabi D, et al. The effects of low protein products availability on growth parameters and metabolic control in selected amino acid metabolism disorders patients. Int J Pediatr Adolesc Med. 2018;5:60-8.

16. Mengler K, Garbade SF, Gleich F, et al. Treatment outcomes for maple syrup urine disease detected by newborn screening. Pediatrics. 2024;154:e2023064370.

17. Magdy RM, Dolins KR, Nagdy H, Ali TM, Elabd HS, Hassan MA. Assessment of quality of life in families affected by maple syrup urine disease: a cross sectional study. J Pediatr Endocrinol Metab. 2025;38:65-72.
