# Supplementary material for: Management of acute metabolic decompensation in maple syrup urine disease: guidance based on international clinical practice
Source: Orphanet J Rare Dis. 2026 Jun 6;21:279. doi: 10.1186/s13023-026-04418-y (PMC13471534; doi:10.1186/s13023-026-04418-y)
Supplement: Supplementary file 2 [file 13023_2026_4418_MOESM2_ESM.pdf]

Lay summary

Maple syrup urine disease (MSUD) is a rare inherited metabolic disease caused by a deficiency in an enzyme called branched-chain ketoacid dehydrogenase (BCKD), which is involved in the pathway that breaks down the branched-chain amino acids (BCAAs) leucine, valine, and isoleucine. The lack of BCKD in MSUD results in high levels of leucine in the blood, which leads to life-threatening brain-related symptoms if left untreated. Patients with MSUD must follow a restricted low-BCAA diet to avoid increasing leucine levels that is supplemented with a BCAA-free mixture to ensure adequate protein intake, but life-threatening episodes of high leucine may still occur. As a result, patients and their families experience high levels of anxiety and poor quality of life. To reduce leucine levels in the blood, patients are administered BCAA-free mixtures either orally, through a nasogastric tube, or intravenously. The use of oral BCAA-free mixtures can be challenging due to gastrointestinal intolerance and because adults tend to refuse nasogastric tubes. Intravenous administration may overcome these issues, but currently there are no commercially available intravenous BCAA-free mixtures. Liver transplantation can prevent life-threatening episodes of high plasma leucine and improve quality of life in patients who are suitable for transplant. Research into newer treatment options for MSUD is ongoing.
